# Effect of feeding mixed microbial culture fortified with trace minerals on ruminal fermentation, nutrient digestibility, nitrogen and trace mineral balance in Sheep

**DOI:** 10.1186/s40781-016-0102-8

**Published:** 2016-05-25

**Authors:** W. S. Kwak, Y. I. Kim, D. Y. Choi, Y. H. Lee

**Affiliations:** Division of Food Biosciences, College of Health and Medical Life Sciences, Konkuk University, Chung-Ju, Chung-Buk 380-701 Korea

**Keywords:** Microbial culture, Trace mineral, Absorption, Excretion, Retention, Sheep

## Abstract

**Background:**

The aim of the present study was to determine the effects of feeding trace mineralsfortified mixed microbial culture (TMC) on ruminal fermentation, nutrient digestibility, blood electrolyte status, nitrogen balance, and trace mineral balance in sheep.

**Methods:**

Mixed microbes [0.6 % (v/w) of *Enterobacter* sp*.*, *Bacillus* sp*.*, *Lactobacillus* sp*.,* and *Saccharomyces* sp*.*] were cultured with 99 % feedstuffs and 0.4 % trace minerals including zinc and copper for ensiling. Six sheep (a mean body weight of 46.5 ± 1.2 kg) were fed two diets: a control diet (concentrate mix and rye straw) and an experimental diet (a control diet + 3.1 % TMC).

**Results:**

TMC feeding did not induce negative effects on ruminal fermentation, nutrient digestibility, blood electrolytes, and nitrogen balance in sheep. Feeding with TMC increased the intake of trace minerals (*p* < 0.05) and did not affect absorption of trace minerals in the whole digestive tract. Feeding with TMC increased fecal excretion and absorbable intake, and retention of zinc and copper (*p* < 0.05) by 71 % and 77 %, respectively.

**Conclusion:**

Feeding with TMC resulted in higher zinc and copper bioavailability and retention without any adverse effects on sheep performance.

## Background

Feeding animals with microbials directly improves ruminal acidosis prevention, gut microbial balance, feed intake, weight gain, and feed efficiency in ruminants [1–3]. For instance, feeding with yeast culture increased feed intake in lactating cows [4, 5] and Hereford steers [6, 7], resulting in better animal performance. The use of microorganisms in animal diets has been shown to improve trace mineral balance. Yeast culture supplementation improved the retention of zinc and iron in lambs [7] and copper and iron in growing ruminants [1]. Feeding with mixed microbial (*Bacillus* and *Saccharomyces*) culture was shown to improve bioavailability of trace minerals (zinc, copper, and iron) to beef steers [8].

Trace mineral deficiency affects almost all physiological processes like growth, reproduction, immunity, milk production and other functions of animals [9]. Dietary trace minerals such as zinc and copper are essential components of enzymes needed for growth and lactation [10]. Fat metabolism seems to be associated with zinc. The glucose incorporation into fatty acids is greatly reduced in zinc-deficient animals [11]. According to previous studies, addition of zinc to cattle diets improves the growth of animals and carcass characteristics [12, 13].

Copper deficiency is an important problem in ruminants in many areas of the world. Copper deficiency frequently occurs because copper antagonists that reduce the bioavailability of copper, such as molybdenum, sulfur, and iron, are often high in ruminant diets [14]. Relatively low dietary concentrations of sulfur and molybdenum can increase copper requirement by 2 to 3-fold [10]. Copper supplementation affected lipid metabolism by reducing cholesterol concentrations in the *longissimus* muscle of Brangus bulls in the study of Netto et al. [15]. Indoor confinement systems of cattle lowered zinc and copper intake from pastures and soils. Zinc and copper supplementation of cattle diets becomes increasingly important with regard to the greater use of high concentrate feeds [11]. In practice, feed manufacturers use higher concentration of zinc and copper than those specified for ruminants to achieve the maximum performance [16]. Zinc and copper supplementation in human food is especially important for pregnant women, and people consuming less animal products rich in zinc and copper [11]. As found in our previous research, feeding a combination of mixed microbial culture and clay minerals to beef steers increased the concentrations of zinc and copper in the *longissimus* muscle [8]. Feeding with trace minerals-fortified mixed microbial culture (TMC)-supplemented diet increased concentrations of certain trace minerals in the *longissimus* muscle of Hanwoo steers [17]. Thus, in the current research, we hypothesized that supplementing a mixed microbial culture with trace minerals (zinc and copper) will positively affect the ruminant performance and still increase bioavailability or retention of zinc and copper when the dietary zinc and copper contents increase from 1.5-fold to 2.5-fold of dietary requirement.

Accordingly, the aim of the study was to evaluate the effects of TMC on nutrient digestibility, ruminal fermentation, blood electrolytes, nitrogen (N) balance, and trace mineral balance of sheep.

## Methods

### Preparation of TMC

The mixed microbial inoculants used in during experiments were isolated from the spent mushroom substrate and identified previously in our lab [18, 19]. Mixed bacteria culture included highly cellulolytic bacteria such as *Enterobacter ludwigii* KU201-3, *Bacillus cereus* KU206-3, and *Bacillus subtilis* KU3, anaerobic fermentation-stimulating lactic acid bacteria such as *Lactobacillus plantarum* KU5, and commercial *Saccharomyces cerevisiae*. Each of these strains was inoculated at 0.12 % of the mixture. *Bacillus* sp*.* and *Enterobacter* sp. were cultured in plate containing count broth (5 g casein, 2.5 g yeast extract, and 1 g/L dextrose) at 36 °C for 24 h. *Lactobacillus* sp*.* was cultured in MRS broth (0881, Difco Laboratories Inc., Detroit, MI, USA) at 36 °C for 24 h. *Saccharomyces* sp. was cultured in yeast malt broth (0711, Difco Laboratories Inc.) at 30 °C for 48 h.

These microbes (0.6 % v/w) were inoculated into the mixture of 24.4 % defatted rice bran, 19.6 % ground corn grain, 16.0 % soybean meal, 20.0 % bentonite, 12.2 % spent mushroom substrate, 3.3 % jujube (low quality), 2.5 % molasses, 1.6 % MgO, and 0.4 % trace minerals, fermented for 5 d, and dried. In order to obtain the treated diet, trace minerals, such as zinc sulfate and copper sulfate, were added to microbial culture in the amount approximately 3-fold higher than the dietary requirement of sheep [10]. The chemical and trace mineral composition of TMC as well as the content of concentrate mix and rye straw fed to sheep are presented in Table 1. The crude protein (CP) and copper content of rye straw were similar to those (3.0 % CP and 4.0 ppm copper) presented in NRC [20]. Zinc concentrations in rye straw were much lower than those in typical cereal grain and pasture herbage (20–30 ppm) [10]. Drinking water from the underground source contained 0.33 ppm iron, 0.02 ppm manganese, 2.8 ppm zinc, 0.65 ppm copper, 0.03 ppm cobalt, and 0.03 ppm molybdenum (data not presented).

**Table 1:** Chemical composition of feedstuffs fed to sheep^a^

Item	Concentrate mix	Rye straw	TMC^b^
Dry matter, %	88.0	87.5	81.7
Organic matter, %	91.8	92.8	68.6
Ether extract, %	2.8	0.2	0.2
Crude protein, %	17.4	3.1	16.1
Neutral detergent fiber, %	31.5	72.5	23.4
Acid detergent fiber, %	16.2	45.7	12.3
Crude fiber, %	11.0	36.5	7.0
Nitrogen free extracts, %	60.7	53.0	45.3
Crude ash, %	8.2	7.2	31.4
Ca, %	1.14	0.26	0.90
P, %	0.58	0.10	0.23
Trace mineral, ppm			
Zn	83.5	9.8	1,149.5
Cu	21.3	4.1	410.1
Co	0.02	0.01	3.59
Fe	884.3	65.9	4,230.5
Mn	130.9	47.0	233.3
Mo	0.29	0.23	nd^c^

^a^On a dry matter basis

^b^TMC = trace mineral-fortified microbial culture

^c^ nd = not detected

The ingredient, chemical, and trace mineral composition of the control and treated diets fed to sheep are presented in Table 2. The TMC-added diet contained zinc in the amount 1.6-fold higher and copper in the amount 1.9-fold higher than that in the control diet.

**Table 2:** Ingredient, chemical, and trace mineral composition of control diet and diet added with trace mineral-fortified microbial culture (TMC)^a^

Items	Control	TMC added
Ingredient composition (g/d)		
Concentrate mix	510.0	510.0
Rye straw	340.0	340.0
Total	850.0	876.1
Chemical composition (%)		
Dry matter	87.8	87.6
Organic matter	92.2	91.5
Crude protein	11.7	11.8
Ether extract	1.8	1.7
Crude fiber	21.2	20.7
Neutral detergent fiber	47.9	47.1
Acid detergent fiber	28.0	27.5
Nitrogen free extracts	57.6	57.2
Crude ash	7.8	8.6
Ca	0.79	0.79
P	0.39	0.39
Trace mineral composition, mg/kg		
Zn	54.0	89.0
Cu	14.4	27.1
Co	0.02	0.11
Fe	557.0	674.3
Mn	97.4	101.7
Mo	0.26	0.25

^a^On a dry matter basis

### Animals, feeding and experimental design

All animal care protocols were approved by the Konkuk University Institutional Animal Care and Use Committee. Six 3-yr old sheep (a mean body weight of 46.5 ± 1.2 kg) were randomly allotted to two dietary treatments as shown in the Table 2: a control diet (formulated concentrate mix and rye straw) and a treated diet (control diet + 3.1 % TMC). Dietary ingredients were mixed daily. Control diet dry matter (DM, approximately 1.8 % of body weight) was fed in equal portions in the amount of 850 g at 07:00 and 18:00 h. TMC was top-dressed on feed for the treated group. The amount of diet DM corresponded to the requirements of sheep [20]. During the experiment, sheep always had free access to fresh water. Diets were randomly assigned to sheep at the start of each trial provided that sheep would not receive the same diet in two consecutive trials. A switch-over design was used in this study. Each trial consisted of 5 d transition, 10 d preliminary, and 7 d collection periods. The number of observations per treatment was 6.

During the experiment, the animals were kept in individual metabolism crate (1.6 m × 0.5 m) that permitted separate collection of feces and urine. Daily fecal output during the collection period was dried at 60 °C. Feces were thoroughly mixed at the end of the collection period to obtain a composite sample, which was ground through a 2 mm screen prior to storage. Daily urine output was collected in plastic bottles containing 15 ml of 13.5 N H_2_SO_4_. After weighing, 2 % of the urine volume was refrigerated and bulked for the collection period.

About 250 ml of ruminal fluid was obtained through the throat. Fluid was strained through four layers of gauges prior to the determination of pH. Five ml of fluid were transferred to a tube containing two drops of concentrated H_2_SO_4_ and another 5 ml to a tube containing 1 ml of 25 % wt/vol of metaphosphoric acid plus 5 ml isocaproic acid for the determination of NH_3_-N and volatile fatty acids (VFA), respectively. Samples were stored at -20 °C. In addition, on the last day of each trial, blood samples were taken via jugular venipuncture 6 h after feeding, added into bottles with anticoagulant ethylenediaminetetraacetic acid, and stored at -20 °C for subsequent analysis.

### Chemical analysis

Representative samples of the test feeds offered to the sheep were collected and stored at -20 °C for further analysis. Immediately before the analysis, all the samples were dried and ground to pass through a 1-mm filter using a sample mill (Cemotec, Tecator, Sweden). The DM fraction was quantified by drying the samples at 60 °C for 48 h until constant weight. The crude protein (CP), ether extract (EE), neutral detergent fiber (NDF), acid detergent fiber (ADF), crude fiber, and crude ash contents were determined following AOAC [21].

Ruminal NH_3_-N was determined according to Chaney and Marbach [22]. Ruminal VFA was determined according to Erwin et al. [23]. For mineral analysis, samples were analyzed for calcium, phosphorus, potassium, sodium, chlorine, zinc, copper, cobalt, iron, manganese, and molybdenum by inductively coupled argon plasma emission spectroscopy (ICP-OES 5300DV, Perkin Elmer, Billerica, Massachusetts, USA) as described by Braselton et al. [24].

### Statistical analysis

Data were subjected to one-way analysis of variance (ANOVA) using the general linear model procedure for a completely random design [25]. The model included diet, trial, and interaction between diet and trial. Interaction effects for all dependent variables (parameters observed) were non-significant (*P* > 0.1), and were removed from the model. A comparison between the mean values of the control and treated diets was made using a student’s t test. [25] The differences were considered statistically significant at *p* < 0.05.

## Results

### Ruminal fermentation, nutrients digestibility, and N balance

Feeding TMC (26.1 g/d) to sheep (850 g/d DM intake for the control group) did not affect ruminal fermentation such as ruminal pH, VFA proportions and total VFA production, and NH_3_-N (Table 3). Feeding TMC to sheep did not affect the apparent digestibility of nutrients (Table 4). For N balance, feeding TMC increased N intake (*p* < 0.05), but did not affect fecal and urinary N excretion, and N retention (Table 5).

**Table 3:** Ruminal parameters of sheep fed fed trace mineral-fortified microbial culture (TMC)

Item	Control	TMC added	SE	*p* value
Ruminal pH	6.41	6.52	0.12	0.3861
Ruminal total VFA^1)^, μmoles/ml	47.1	47.2	8.9	0.9949
Moles/100moles				
Acetate	72.2	73.1	1.2	0.4963
Propionate	17.8	16.7	2.0	0.6047
* Iso*-butyrate	0.22	0.22	0.02	0.8417
Butyrate	9.32	9.50	1.32	0.8891
* Iso*-valerate	0.19	0.22	0.05	0.5152
Valerate	0.25	0.27	0.04	0.6671
Caproic acid	0.02	0.03	0.01	0.6746
Acetate/Propionate	4.12	4.63	0.51	0.3421
Ruminal NH_3_-N, mg/dl	12.8	12.5	1.7	0.8629

^1)^ Volatile fatty acids

^a,b^Means with different superscripts within the same row are significantly different (*p* < 0.05)

**Table 4:** Apparent digestibility of nutrient by sheep fed trace mineral-fortified microbial culture (TMC)^1)^

Item	Control	TMC added	SE	*p* value
	--------------- % ---------------			
Dry matter	67.8	67.8	0.7	0.9427
Organic matter	70.1	70.5	0.8	0.5988
Ether extract	86.0	86.1	1.9	0.9612
Crude protein	67.7	68.1	1.8	0.8240
Neutral detergent fiber	58.8	58.2	1.5	0.7157
Acid detergent fiber	52.4	52.4	2.3	0.9924
Hemicellulose	67.7	66.3	1.5	0.3879
Crude fiber	51.3	51.5	2.0	0.9225
Nitrogen free extracts	77.0	77.5	0.8	0.5250
Crude ash	40.2	38.5	1.6	0.3194
Total digestible nutrient, %	66.5	66.4	0.7	0.8674

^1)^On a dry matter basis

^a,b^Means with different superscripts within the same row are significantly different (*p* < 0.05)

**Table 5:** Nitrogen balance of sheep fed trace mineral-fortified microbial culture (TMC)^1)^

Item	Control	TMC added	SE	*p* value
Intake, g/d	15.9^b^	16.5^a^	0.1	0.0001
Excretion, g/d				
Fecal	5.1	5.3	0.3	0.6181
Urinary	8.9	8.8	0.6	0.8203
Total	14.0	14.0	0.5	0.9656
Absorption, g/d	10.7	11.3	0.3	0.1203
Retention				
g/d	1.8	2.5	0.5	0.1928
% intake	11.6	15.0	2.8	0.2387
% absorbed	17.1	22.2	4.1	0.2374

^1)^ On a dry matter basis

^a,b^Means with different superscripts within the same row are significantly different (*p* < 0.05)

### Trace mineral intake and apparent absorption

Intake of almost all the trace minerals except for Mo increased (*p* < 0.05) in sheep fed with treated diet, because TMC was supplemented with zinc and copper and contained inherent cobalt, iron and manganese (Table 6). Apparent absorption of trace minerals was not affected by TMC feeding.

**Table 6:** Intake, absorbable intake and apparent absorbability of trace minerals by sheep fed trace mineral-fortified microbial culture (TMC)^1)^

Item	Control	TMC added	SE	*p* value
Intake, mg/d				
Zn	47.4^b^	77.2^a^	0.2	<0.0001
Cu	12.6^b^	23.3^a^	0.1	<0.0001
Co	0.02^b^	0.11^a^	0.01	<0.0001
Fe	473.6^b^	584.0^a^	0.1	<0.0001
Mn	82.8^b^	88.9^a^	0.1	<0.0001
Mo	0.24	0.24	0.01	0.3974
Absorbable intake, mg/d				
Zn	8.7^b^	14.9^a^	2.0	0.0363
Cu	2.33^b^	3.96^a^	0.24	0.0088
Co	0.001	0.002	0.03	0.5500
Fe	120.8	137.8	24.7	0.5264
Mn	-1.24	-0.44	1.37	0.5867
Mo	0.057	0.053	0.030	0.9237
Apparent absorbability, %				
Zn	18.4	19.3	3.4	0.8077
Cu	18.5	17.0	1.5	0.3956
Co	1.7	2.1	5.6	0.5544
Fe	25.5	23.6	5.1	0.7312
Mn	-1.5	-0.5	1.6	0.5597
Mo	23.3	22.4	12.3	0.9433

^1)^On a dry matter basis

^a,b^Means with different superscripts within the same row are significantly different (*p* < 0.05)

### Trace mineral excretion and retention

Followed by the increased intake of trace minerals, TMC feeding increased fecal excretion of zinc, copper, cobalt, iron, and manganese (*p* < 0.05) (Table 7). Urinary excretion of trace minerals was not different between the treatments. TMC feeding increased daily zinc and copper retention (*p* < 0.05), and did not affect other trace mineral retention (Table 7).

**Table 7:** Trace mineral excretion and retention of sheep fed trace mineral-fortified microbial culture (TMC) ^1)^

Item	Control	TMC added	SE	*p* value
	--------- mg/d --------			
Fecal excretion				
Zn	38.7^b^	62.3^a^	2.1	0.0004
Cu	10.3^b^	19.3^a^	0.3	0.0001
Co	0.0^b^	0.1^a^	0.1	0.0412
Fe	352.7^b^	446.0^a^	24.7	0.0195
Mn	84.0^b^	89.3^a^	1.4	0.0180
Mo	0.2	0.2	0.1	0.9818
Urinary excretion				
Zn	nd^2)^	nd	-	-
Cu	0.09	0.05	0.10	0.6792
Co	nd	nd	-	-
Fe	nd	nd	-	-
Mn	nd	nd	-	-
Mo	0.09	0.05	0.03	0.2302
Retention				
Zn	8.7^b^	14.9^a^	2.0	0.0363
Cu	2.2^b^	3.9^a^	0.3	0.0048
Co	0.011	0.015	0.020	0.9900
Fe	120.8	138.0	24.7	0.5264
Mn	-1.2	-0.4	1.4	0.5867
Mo	-0.036	-0.007	0.057	0.4903

^1)^ On a dry matter basis. ^2)^ nd = not detected

^a,b^Means with different superscripts within the same row are significantly different (*p* < 0.05)

In addition, TMC feeding did not affect blood electrolytes such as calcium, phosphorus, potassium, sodium, and chlorine (data not presented). It indicates that TMC feeding maintained normal metabolism and homeostasis of major minerals in the body.

## Discussion

### Rumen fermentation, nutrients digestibility, and N balance

Feeding TMC to sheep did not affect ruminal fermentation, apparent digestibility of nutrients, and nitrogen balance in sheep. In spite of the high ash content (31.4 %) in TMC, the diet treated with TMC appeared to contain similar total digestible nutrients as the control diet. This finding suggests that feeding TMC positively affects nutrient digestion of the control (basal) diet. In previous studies, feeding direct-fed microbials (*Enterococcus* sp. and yeast sp.) to feedlot cattle affected ruminal fermentation and nutrient digestion through the decrease of ruminal pH and butyrate, and an increase of propionate [26]. Feeding *Saccharomyces* sp. and/or *Clostridium* sp. increased the total ruminal VFA concentration, acetic acid proportion, and nutrient digestibility, and decreased propionate proportion in sheep [27]. Feeding ruminal cellulolytic bacteria culture to Hanwoo heifers increased concentration of ruminal butyrate and NH_3_-N but did not affect ruminal pH and concentrations of ruminal total VFA, acetate, propionate and valerate [28]. Such inconsistent results suggest that ruminal fermentation and nutrient digestibility vary depending on the species and the amount of microbes added to the diet.

Supplementing 20 mg/kg of zinc sulfate to the diet did not affect ruminal total VFA concentration and proportions [29]. According to Fathul and Wajizah [30], the addition of copper and manganese does not alter *in vitro* ruminal pH, NH_3_-N, and VFA. Similarly, mineral compounds did not affect ruminal pH, VFA, and NH_3_-N in the study of Galyean and Chabot [31]. These results consistent between studies suggest that trace minerals have little effect on ruminal fermentation.

According to the study of Cole et al. [7], lambs fed yeast culture had greater N balance than control lambs. This phenomenon was not observed in the present study.

### Intake and apparent absorption of trace minerals

The intake of zinc and copper by sheep fed with treated diet increased by 63 and 89 %, respectively (*p* < 0.05). An apparent absorption of trace minerals was similar between sheep fed with TMC and control diets. This means that the supplementation of trace minerals did not decrease the apparent absorption of these minerals in sheep diet. As a result, absorbable intake of zinc and copper increased by 71 and 70 %, respectively. The apparent absorption of zinc (18–19 %) belonged to the generally common range, 15–60 % as reported by McDowell [11]. High intake of divalent cations, such as copper, iron, and calcium, and phytate reduce zinc absorption [10]. The most important factor affecting zinc absorption is the zinc content of the diet [11]. In the present study, zinc supplementation at the marginal, not deficient dietary level, did not alter zinc absorption percentage.

Copper absorption (17 - 18 %) in the present study was higher than that (<1.0 to 10 percent) in ruminants [14]. Copper has complex interactions with sulfur and molybdenum in the ruminal environment. High dietary zinc and iron can reduce copper absorption in cattle and sheep [14, 32, 33]. In the present study, copper supplementation at the marginal, not deficient dietary level, did not alter copper absorption percentage, thus resulting in increased absorbable intake of copper.

Absorption of cobalt as found in the current study was 1.7–2.1 % and fell in the range 1–2 % as reported by Van Bruwaene et al. [34]. Absorption of iron and molybdenum was positive as reported by Ben-Ghedalia et al. [35]. However, apparent absorption of manganese was negative as reported by Solomon et al. [36] in sheep.

### Trace mineral excretion

Fecal excretion of zinc and copper in sheep was higher by 61 and 87 % respectively at TMC diet than control diet. The primary route of trace mineral excretion was through the feces rather than urine. Similar result was also observed for zinc. The primary route of zinc excretion was through the feces [37]. As observed in the present study, the urinary excretion of zinc by sheep was generally less than 1 mg/d with little effect on excretion due to zinc supply in the diet [10]. Copper also showed similar excretion pattern to zinc. As reported in previous studies, biliary excretion is the major mechanism responsible for copper homeostasis, which is less effective in sheep than cattle [38].

Absorbed cobalt is primarily excreted though the urine [39]. Cobalt concentrations were extremely low and not detectable in the present study. The primary routes of iron excretion are via feces and urine [11]. However, in the present study the fecal route appeared to be more important. Excretion of absorbed manganese in the bile occurs very rapidly [40]. In the present study, more endogenous manganese seemed to excrete via the feces than supplied manganese. Molybdenum was excreted through both the feces and urine. The results of present study are in agreement with the report of NRC [10] showing that feces serve major excretion pathway in ruminants, and body balance of molybdenum is controlled primarily by urinary excretion.

### Trace mineral retention

Zinc retention and copper retention in sheep were higher by 71 and 77 %, respectively at TMC diet than that at the control diet. This phenomenon was observed consistently in previous studies, too. Supplementing yeast culture increased retention of zinc or copper in lambs [7, 41]. Feeding a combination of mixed microbial culture and clay minerals to beef steers increased concentrations of zinc and copper in the *longissimus* muscle [8]. Cao et al. [42] reported that zinc supplementation of ruminant diets deficient in zinc resulted in the increased zinc retention in the ruminant muscle. Supplementing zinc and other trace minerals increased concentrations of these minerals in the *longissimus* muscle of Hanwoo steers [7]. When the dietary zinc concentration was lower than 100 ppm diet, the tissues of lambs did not accumulate zinc, but muscle zinc was higher at 100 ppm zinc level than at 50 ppm zinc level [43]. Retained zinc primarily accumulates in skeletal muscles and bones, as also suggested by Outten and O’Halloran [44]. The copper is mainly stored in liver. Increasing dietary copper at the deficient or marginal level does not increase the copper concentration in muscles [10]. In this study, higher amount of retained copper was expected to be stored primarily in liver.

Increased intake of cobalt did not result in higher cobalt retention, apparently due to higher fecal excretion. This observation may explain why supplemented cobalt did not accumulate in the *longissimus* muscle of Hanwoo steers in the study of Kwak et al. [17]. Henry et al. [45] reported that liver and kidney cobalt concentrations increase when animals are fed high dietary cobalt. However, this phenomenon was not observed in the current study.

The overall results of the present study indicate that a desirable trace mineral retention in sheep may happen when a diet is supplemented with both microbial culture and trace minerals. This phenomenon can be explained by an improved mineral uptake through the microbial bioconversion of inorganic trace minerals into more available organic minerals that may occur during the ensiling process and the ruminal fermentation process. To summarize, TMC feeding resulted in higher intake of trace minerals (especially Zn and Cu), similar absorption, higher absorbable intake, higher fecal and similar urinary excretion, and higher retention.

## Conclusions

The results of the current study suggest that supplementing a combination of mixed microbial culture and trace minerals improves the bioavailability and retention of zinc and copper in sheep without adverse effects on ruminal fermentation, nutrient digestibility, blood electrolytes, and nitrogen balance. The results of the present research can be used as a basis for producing healthier animal feed and animal products rich with certain trace minerals.
